# Variations in Concentration and Carbon Isotope Composition of Methanotroph Biomarkers in Sedge Peatlands Along the Altitude Gradient in the Changbai Mountain, China

**DOI:** 10.3389/fmicb.2022.892430

**Published:** 2022-05-18

**Authors:** Meiling Zhao, Ming Wang, Yantong Zhao, Ming Jiang, Guodong Wang

**Affiliations:** ^1^Key Laboratory of Wetland Ecology and Environment, Northeast Institute of Geography and Agroecology, Chinese Academy of Sciences, Changchun, China; ^2^College of Resources and Environment, University of Chinese Academy of Sciences, Beijing, China; ^3^State Environmental Protection Key Laboratory of Wetland Ecology and Vegetation Restoration, Institute for Peat and Mire Research, Northeast Normal University, Changchun, China

**Keywords:** altitude gradient, methanotroph biomarker, metabolic characteristics, sedge peatland, competitive distribution

## Abstract

Northern peatlands are one of the largest natural sources of atmospheric methane globally. As the only biological sink of methane, different groups of methanotrophs use different carbon sources. However, the variations in microbial biomass and metabolism of different methanotrophic groups in peatlands along the altitude gradient are uncertain. We measured the concentrations and metabolic characteristics of type I (16:1ω7c and 16:1ω5c) and type II (18:1ω7c) methanotroph biomarkers using biomarkers and stable isotopes in eight *Carex* peatlands along an altitude gradient from 300 to 1,500 m in the Changbai Mountain, China. We found that the trends with altitude in concentrations of the type I and type II methanotroph biomarkers were different. The dominating microbial group changed from type I to type II methanotroph with increasing altitude. The concentrations of type I and type II methanotroph biomarkers were significantly affected by the total phosphorus, total nitrogen, and dissolved organic carbon, respectively. The δ^13^C values of type II methanotroph biomarkers changed significantly along the altitude gradient, and they were more depleted than type II methanotroph biomarkers, which indicates the difference in carbon source preference between type I and type II methanotrophs. This study highlights the difference in the concentration and carbon source utilization of type I and type II methanotrophic groups along the altitude gradient, and enhances our understanding of the metabolic process of methane mediated by methanotrophs and its impact on carbon-sink function in northern peatlands.

## Introduction

It is predicted that wetlands will form the majority of methane (CH_4_) climate feedback by 2,100 (Dean et al., 2018). Peatlands represent large terrestrial carbon banks, which are also one of the largest natural sources of CH_4_ emissions into the atmosphere (Chowdhury and Dick, 2013). Even a very small change in CH_4_ emissions in peatlands will have a strong impact on the trajectory of future global climate change, so the metabolic process of CH_4_ in peatlands has attracted considerable attention (Chowdhury and Dick, 2013; Zhao et al., 2021). As the only biological sink of CH_4_, the methanotrophs use CH_4_ as the source of carbon and energy, so that the largest part of CH_4_ formed in peatland ecosystems is recycled and does not reach the atmosphere (Raghoebarsing et al., 2005; Kip et al., 2011). Understanding the biogeochemical processes mediated by methanotrophs in peatlands is the key in predicting future climate change.

Methanotrophs can be divided into two groups based on differences in phenotypic, chemotaxonomic classifications, and genotypic characteristics, such as the arrangement of cytoplasmic intima, dominant phospholipid fatty acids (PLFAs), and carbon assimilation pathways. The two types are: types I and II methanotrophs, which belong to the Gamma- and Alphaproteobacteria, respectively (Dedysh et al., 2007). The PLFAs are the main components of the membranes of all living cells. They also record the substrate-organic matter information (Zhao et al., 2020). The results of PLFAs combined with natural ^13^C content can be used to interpret the functions of microbial groups in soils (Watzinger, 2015). The carbon isotopic composition of the methanotrophs was more depleted than that of other microbial groups in peatlands. This is because the methanotrophs use CH_4_ with a significantly negative δ^13^C value as a carbon source, and significant carbon isotope fractionation will occur in the process of assimilation and absorption of CH_4_ (Cifuentes and Salata, 2001; Zhang et al., 2021). Due to the difference in metabolic characteristics and morphology between type I and type II methanotrophs, they might also differ in their values of δ^13^C. However, little is known about the δ^13^C characteristics of type I and type II methanotrophs. Such knowledge is very important for the understanding of the biogeochemical processes mediated by methanotrophs in peatlands.

The altitude gradient was supposed to be an ideal natural laboratory for studying climate change (Siles et al., 2017). The peatlands along the altitude gradient are important ecosystems in the Changbai Mountain regain of China, and they are extremely sensitive to climate change (Wang et al., 2021; Zhao et al., 2021). Therefore, we selected eight sedge-dominated peatlands along an altitude gradient in the Changbai Mountain to reveal the influence of the environmental changes along an altitude gradient on the concentrations and metabolic characteristics of methanotrophs in peatlands. We asked the following research questions: (1) how does the concentration of type I and type II methanotroph biomarkers change along the altitude gradient? (2) If the trends in δ^13^C values of type I and type II methanotrophs differ along the altitude gradient?

## Materials and Methods

### Study Area

We collected samples on the north slope of the Changbai Mountain (41°41′49″– 42°25′18″ N, 127°42′55″–128°16′48″ E). Changbai Mountain is located in the northeast of China and is adjacent to the border between China and North Korea. Changbai Mountain is the highest mountain on the eastern edge of Eurasia, and it is vulnerable to climate change (Wang et al., 2021; Zhao et al., 2021). The peatlands are widely distributed in the Changbai Mountains with an altitude of about 300 to 1,500 m. The total area of peatlands in this region is about 23,100 ha, of which 70% are sedge peatlands dominated by *Carex* species (Bao et al., 2010; Wang et al., 2017). This area belongs to a temperate continental monsoon climate, which is characterized by long, cold winters and short, cool summers. The average annual temperature (MAT) from 327 to 1,485 m in our study ranges from –0.175 to 3.3°C, and the annual precipitation (MAP) is between 605 and 766 mm.

### Soil Sampling

**FIGURE 1:**
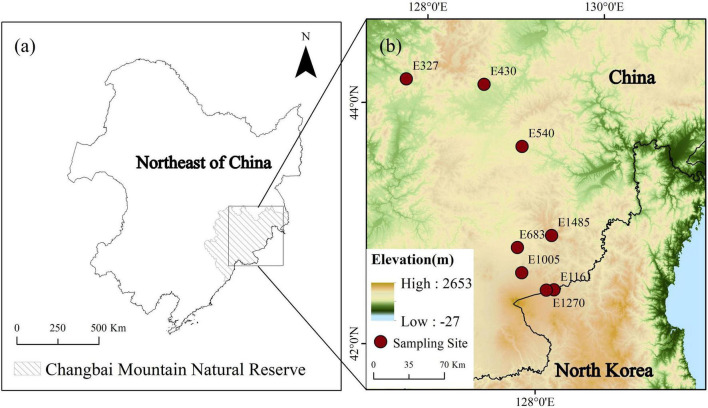
Locations of the sampling sites along the altitude gradient in peatlands of the Changbai Mountain, China.

In July 2020, we collected soil samples of the top 5 cm in 8 sedge peatlands along an altitude gradient (327, 430, 540, 683, 1,005, 1,161, 1,270, and 1,485 m; Figure 1; and Table 1). These sites were all dominated by *Carex schmidtii*, which is a tussock-forming sedge species (Zhao et al., 2021). All the sites were flooded during the growing season. The sampling sites were located in the hollows with a 2–5 cm water depth (Wang et al., 2017). Three replicate samples were collected at each study site. The location of each sample was at least 10 m apart from each other. The peat samples were stored at –20°C.

**TABLE 1:** Site information and general climate and soil characteristics in the sedge-dominated peatlands of the Changbai Mountains, China.

		MAT	MAP		TOC		SWC	DOC	TP	TN
Site name	Site location	(°C)	(mm)	pH	(%)	C/N	(%)	(mg/L)	(mg/g)	(mg/g)
E327	N 44° 06′; E 127°33′	3.3	637	5.1 ± 0.1 b	31.4 ± 0.5	25.4 ± 2.2 ab	80.2 ± 4.0	88.8 ± 3.3 b	1.1 ± 0.5	4.2 ± 0.7
E430	N 43° 52′; E 128° 25′	3.2	605	5.3 ± 0.1 ab	32.4 ± 1.5	23.2 ± 1.5 ab	90.4 ± 0.9	100.0 ± 12.6 b	5.5 ± 2.5	7.1 ± 1.2
E540	N 43° 16′; E 128° 38′	3.25	607	5.1 ± 0.1 b	38.1 ± 0.5	25.4 ± 2.0 ab	85.6 ± 2.9	65.2 ± 13.0 b	3.2 ± 2.3	5.6 ± 0.6
E683	N 42° 27′; E 128° 15′	3.24	671	5.2 ± 0.1 ab	36.9 ± 1.7	26.5 ± 2.0 ab	89.3 ± 0.3	139.5 ± 23.3 b	1.1 ± 1.1	3.8 ± 2.2
E1005	N 42° 14′; E 128° 13′	2.21	706	5.0 ± 0.1 b	31.3 ± 0.5	32.5 ± 3.0 a	83.2 ± 2.6	137.1 ± 9.2 b	1.5 ± 1.2	5.7 ± 3.0
E1161	N 42° 1′; E 128° 31′	1.36	732	6.0 ± 0.1 a	31 ± 0.5	25.7 ± 1.4 ab	81.9 ± 1.5	118.7 ± 13.5b	3.9 ± 2.8	3.7 ± 1.2
E1270	N 42° 2′; E 128° 26′	0.96	743	5.3 ± 0.2 ab	34.7 ± 1.3	24.0 ± 1.4 ab	86.3 ± 1.7	92.8 ± 23.5 b	0.8 ± 0.7	2.5 ± 0.9
E1485	N 42° 28′; E 128° 40′	−0.175	766	4.7 ± 0.1 b	29.5 ± 2.0	21.9 ± 0.8 b	93.1 ± 1.1	213.2 ± 1.8 a	0.5 ± 0.1	3.0 ± 1.0

*MAT, mean annual temperature; MAP, mean annual precipitation; TOC, soil total organic carbon; C/N, ratio of soil carbon to nitrogen; SWC, soil water content; DOC, dissolved organic carbon; TP, total phosphorus; TN, total nitrogen. Data are means ± S.D. (n = 3). Different letters within each column indicate significant differences among study sites based on one-way ANOVA and Tukey’s test (p < 0.05).*

### Soil Physical and Chemical Properties

The soil water content (SWC) was determined by the gravimetric method with the samples freeze-dried (Li et al., 2018). The dissolved organic carbon (DOC) was measured by the method of K_2_SO_4_ extraction with distilled water and 0.5 M K_2_SO_4_ at a 1:5 w/v ratio for 1 h at 20°C (Jones and Willett, 2006). Total phosphorus (TP) and total nitrogen (TN) were measured by the Kjeldahl method and the Molybdenum blue method using an automated analyzer (Smartchem 140, AMS-Alliance, French), respectively. Soil pH was determined by a potentiometric test. The total organic carbon (TOC) and carbon to nitrogen ratio (C/N) were detected on an elemental analyzer (Vario Macro cube, Hanau, Germany).

### The Phospholipid Fatty Acids and the Stable Carbon Isotope Analysis

The analysis of type I and type II methanotrophs was based on PLFA technology. The extraction and separation methods of PLFAs are referred to as the Bligh Dyer method (Bligh and Dyer, 1959; Yao et al., 2012). PLFA composition was identified and analyzed on an Agilent 7890b gas chromatograph (GC) equipped with a 19091b-102 column (25 m × 0.20 mm × 0.33 μm film thickness), and cy19:0 fatty acid methyl ester was used as the external standard. The inlet temperature was 250°C and the detector temperature was 320°C. The carrier gas was helium and the column flow rate was 1.2 ml/min. The heating procedure was an initial temperature of 190°C for 1 min; followed by 10°C/min to 285°C, and finally 60°C/min to 310°C, held for 2 min. The detected compounds were automatically compared with the MIDI library to determine the carbon chain structure of fatty acids (Zhang et al., 2019). The PLFAs of 16:1ω7c and 16:1ω5c were used as the biomarkers of type I methanotroph, and the PLFA of 18:1ω7c was used as the biomarker of type II methanotroph (Bowman et al., 1991, 1993).

The δ^13^C values of PLFAs components were determined using a Finnigan Delta Plus XP instrument equipped with a DB-5MS column (60 m × 0.25 mm × 0.25 μm film thickness), using CO_2_ as a reference gas. The temperatures of the inlet and the detector were 200 and 350°C, respectively. The oxidation furnace temperature was 950°C and the reduction temperature was 650°C. The carrier gas was helium, and the column flow rate was 1.2 ml/min. The heating program had four stages: (1) keeping 100°C for 1 min, (2) 20°C/min to 190°C, (3) 1.5°C/min to 235°C, and (4) 20°C/min to 295°C and holding for 15 min. The Pee Dee Belemnite (PDB) standard was used to report all results of the ^13^C/^12^C ratio in δ notation, expressed in difference per mil (‰): δ^13^C (‰) = [(Rcompounds/Rstd-1)] × 1,000, where Rcompounds was the ratio of ^13^C/^12^C between the samples, and Rstd was the ratio of ^13^C/^12^C in the PDB standard (Börjesson et al., 2016). During methylation, an additional C atom was added to the fatty acid molecule, and the δ^13^C values have been calibrated by formula (Williams et al., 2006).

### Data Analysis

The differences in soil environmental variables, the concentrations, and the δ^13^C values of type I and type II methanotroph biomarkers were performed by one-way ANOVA and a subsequent Tukey’s test. Pearson’s correlation analysis was used to study the relationships between the environmental variables and the concentrations of type I and type II methanotroph biomarkers with the altitude. General linear regression was used to examine the trends in concentrations of type I and type II methanotroph biomarkers along with the altitude. All variables were examined for the normality and homogeneity of variance before analysis. The analysis was carried out in SPSS 21.0. Redundancy analysis (RDA) was used to determine the relationship between type I (16:1ω7c and 16:1ω5c) and type II (18:1ω7c) methanotroph biomarkers and environmental factors (MAT, MAP, TOC, DOC, pH, TN, TP, C/N, and altitude) by using CANOCO 5.

## Results

### Climate and Soil Environmental Factors Along the Altitude Gradient

In the study area, the MAT and MAP varied from 3.3 to –0.175°C and from 605 to 766 mm, respectively (Table 1). The pH ranged from 4.7 to 6.0. The TOC ranged from 29.5 to 38.1%. The TP and TN were highest at E430 (5.5 and 7.1 mg/g), and lowest at sites of E1485 and E1270, respectively. The DOC varied significantly from 88.8 mg/L at E327 to 213.2 mg/L at E1485. The C/N varied significantly from 21.9 at E1485 to 32.5 at E1005. The SWC ranged from 80.2 to 93.1%. There was no significant difference between TOC and SWC across the study sites (Table 2). Pearson’s correlation analysis showed that the altitude was negatively related to MAT, TN, and TP, and it was positively related to DOC (Table 3). There were no significant correlations between the altitude and MAP, TOC, SWC, pH, and C/N (Table 3).

**TABLE 2:** A summary of analysis of variance (one-way ANOVA) on the effect of the altitude for soil environmental variables, and the concentrations of type I and type II methanotroph biomarkers.

Variable	Sum of squares	df	Mean square	*F*	Sig.
Concentration of type I methanotroph biomarker	367.6	7	52.5	7.357	<0.001***
Concentration of type II methanotroph biomarker	488.9	7	69.8	6.052	0.001***
TOC	198.5	7	28.4	1.456	0.252
DOC	43396.3	7	6199.5	6.289	0.001***
SWC	311.2	7	44.5	2.571	0.056
pH	3.1	7	0.4	4.246	0.008**
TN	67.6	7	9.7	2.57	0.048*
TP	59.3	7	8.5	2.977	0.034*
C/N	210.2	7	30.0	2.926	0.036*

TOC, soil total organic carbon; DOC, dissolved organic carbon; TN, total nitrogen; TP, total phosphorus; C/N, carbon and nitrogen ratio. ***p < 0.001, **p < 0.01, *p < 0.05.

**TABLE 3:** Pearson correlation coefficients between the concentrations of type I and type II methanotroph biomarker, environmental variables and the altitude.

	Variables	Correlation coefficient	Sig.
No significant correlations	MAP	0.047	0.829
	TOC	−0.242	0.255
	SWC	0.105	0.625
	pH	0.038	0.858
	C/N	−0.071	0.742
Negative correlations	Concentration of type I methanotroph biomarker	−0.447	0.029*
	MAT	−0.957	<0.001***
	TN	−0.555	0.002**
	TP	−0.445	0.029**
Positive correlations	Concentration of type II methanotroph biomarker	0.488	0.015*
	DOC	0.558	0.005**

MAT, mean annual temperature; MAP, mean annual precipitation; TOC, soil total organic carbon; DOC, dissolved organic carbon; TN, total nitrogen; TP, total phosphorus; C/N, carbon and nitrogen ratio. ***p < 0.001, **p < 0.01, *p < 0.05.

### The Concentration of Methanotroph Biomarkers Along the Altitude Gradient

**FIGURE 2:**
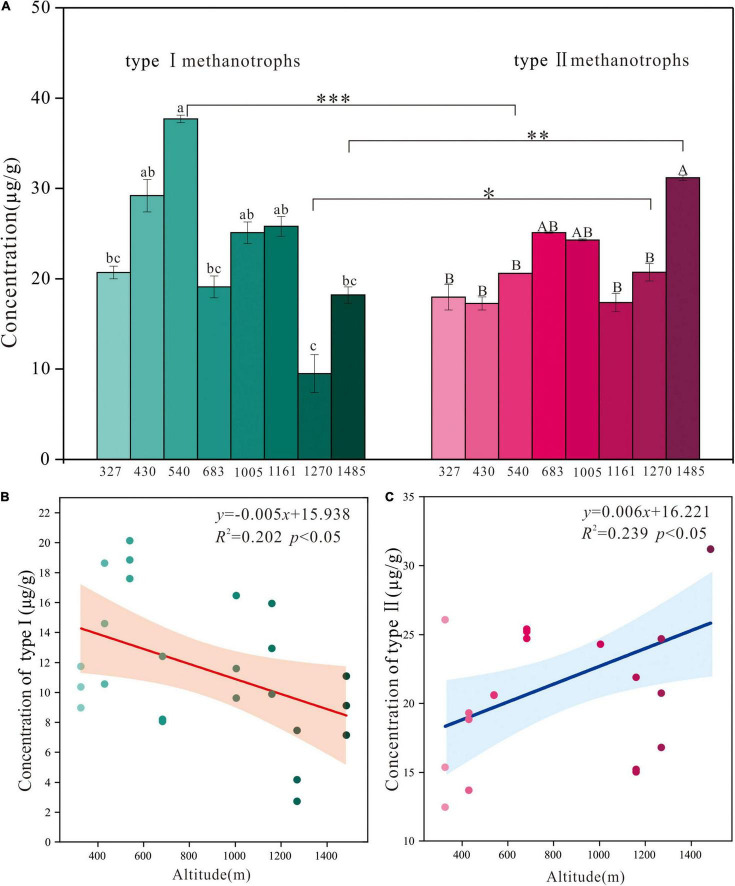
**(A)** The concentrations of type I and type II methanotroph biomarkers along the altitude gradient on the northern slope of the Changbai Mountain, China. Values were presented as means ± S.E. (*n* = 3). Different letters indicate significant differences among study sites based on one-way ANOVA and Tukey’s test (*p* < 0.05). The asterisk represents the difference between type I and type II methanotrophs at the same site (^***^p < 0.001, ^**^p < 0.01, and *p < 0.05). Regression of the altitude against the concentration of type I **(B)** and type II **(C)** methanotroph biomarkers. The R^2^ and *p* values of the regression equation are shown in each panel. Significant relationships are denoted with solid lines, and shadow areas indicate the 95% confidence interval (*CI*) of the fit.

The concentrations of type I and type II methanotroph biomarkers changed significantly along the altitude gradient (Figure 2A). The concentration of the type I methanotroph biomarker was highest at E540 (37.7 μg/g) and lowest at E1270 (9.5 μg/g). The concentration of the type II methanotroph biomarker was highest at E1485 (31.2 μg/g) and lowest at E430 (17.3 μg/g). The concentrations of the two methanotroph biomarkers differed significantly at sites of E540, E1485, and E1270 (Figure 2A). The concentration of type I methanotroph biomarker showed a negative linear relationship with the altitude (Figure 2B), and the concentration of type II methanotroph biomarker showed a positive linear relationship with the altitude (Figure 2C). The Pearson correlation analysis showed that the altitude was negatively related to the concentration of type I methanotroph biomarker, and it was positively related to the concentration of type II methanotroph biomarker (Table 3).

**FIGURE 3:**
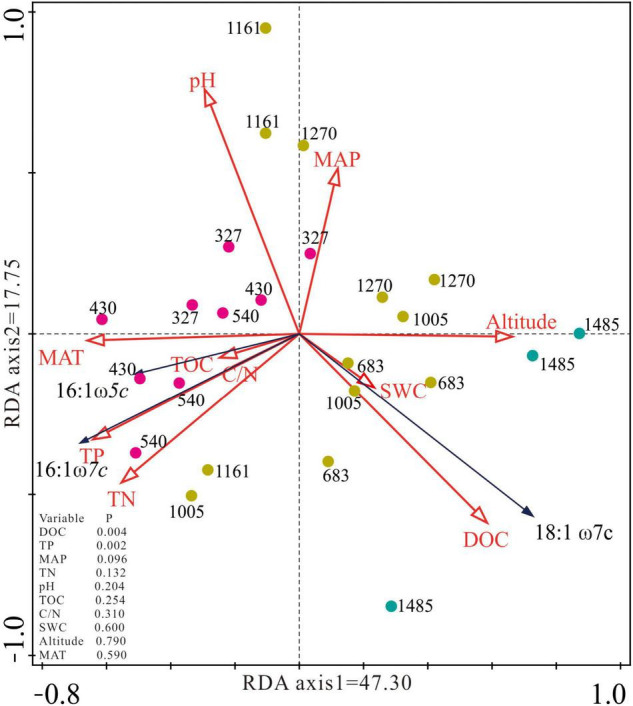
Redundancy analysis (RDA) ordination plot of the concentration of 16:1ω7c, 16:1ω5c, and 18:1ω7c constrained by the altitude, climate, and soil environmental factors. MAT, mean annual temperature; MAP, mean annual precipitation; TOC, soil total organic carbon; DOC, dissolved organic carbon; TN, total nitrogen; TP, total phosphorus; C/N, carbon and nitrogen ratio.

Redundancy analysis showed that the variation in type I (16:1ω5c and 16:1ω7c) and type II (18:1ω7c) methanotroph biomarkers explained by the first axis was 47.30, and 17.75% by the second axis (Figure 3). The environmental change along the altitude gradient was reflected along the first axis of the RDA ordination where the sites in the lower altitude were arranged from high MAT, TN, and TP on the left toward high DOC and SWC in the higher altitude on the right (Figure 3). PLFAs of 16:1ω5c and 16:1ω7c were distributed in the lower left quadrant, while 18:1ω7c was distributed in the lower right quadrant. DOC and TP explained most variation in the concentration of the type I and type II methanotroph biomarkers with the explained degree of 22.3 and 23.4%, respectively (Figure 3). The study sites of E327, E430, and E540 were distributed in the middle left quadrat, the sites of E683, E1005, E1161, and E1270 were mainly distributed in the middle right quadrat, and the site of E1485 was distributed in the lower right quadrat (Figure 3).

### The δ^13^C Values of Methanotroph Biomarkers Along the Altitude Gradient

**FIGURE 4:**
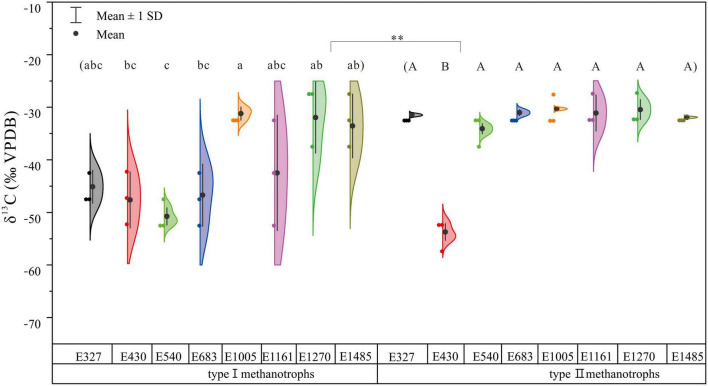
The δ^13^C values of type I and type II methanotroph biomarkers based on phospholipid fatty acids along the altitude gradient on the northern slope of the Changbai Mountain, China. Different letters indicate significant differences among study sites based on one-way ANOVA and Tukey’s test (*p* < 0.05). The asterisk represents the difference between type I and type II methanotrophs (^**^p < 0.01). The δ^13^C values of type I methanotroph biomarkers were the average δ^13^C values of 16:1ω7c and 16:1ω5c.

The δ^13^C values differed significantly between type I and type II methanotroph biomarkers (Figure 4). The δ^13^C values of type I methanotroph biomarkers ranged widely across each site, except for the study sites of E540 and E1005. The δ^13^C values of type I methanotroph biomarkers also changed significantly along the altitude gradient (Figure 4). At the same time, the δ^13^C values of type II methanotroph biomarkers had a small range (Figure 4). The δ^13^C values of type I methanotroph biomarkers were more depleted than type II methanotroph biomarkers. Furthermore, the δ^13^C value of type II methanotroph biomarkers at E430 was more depleted compared with the other seven study sites, but there was no significant difference between these seven sites (*p* > 0.05; Figure 4). Except for E430, the δ^13^C values of type I methanotroph biomarkers were most depleted (–53.72‰) at E1161 among all the sites, and the δ^13^C values of type II methanotroph biomarkers were most depleted (−34.62‰) at E1161 (Figure 4).

## Discussion

### Factors Affecting the Concentration of Methanotroph Biomarkers

In recent years, ecologists have tried to understand patterns in soil microbes and the associated processes along environmental gradients. For example, former researchers had studied the changes in CH_4_ oxidation rate under different conditions, mainly including water table, temperature, vegetation, wildfire, and season (Kim et al., 2011; Danilova et al., 2015). However, little is known about the patterns and functions of the methanotrophs along the altitude gradient and their response to environmental changes in peatlands.

In this study, we found that the concentration of type I methanotroph biomarker was negatively correlated with the altitude, while the concentration of type II methanotroph biomarker was positively correlated with the altitude (Figure 2 and Table 3), which may be caused by the change of peatland environments along with the altitude. The RDA analysis showed that the concentration of type I methanotroph biomarkers (16:1ω7c and 16:1ω5c) was mainly related to TP and TN. In this study, the altitude was negatively related to TN and TP (Table 3), so the TP and TN generally decreased as the altitude increased. Similar to our findings, other studies in paddy fields and forests found that the increase of TN and TP promoted the growth of type I methanotrophs, while type II methanotrophs were generally inhibited (Mohanty et al., 2006; Alam and Jia, 2012). The concentration of type II methanotroph biomarkers was positively correlated with DOC (Figure 3). This is because DOC is more active in the peatland carbon pool and is easily absorbed and utilized by microorganisms, especially the type II methanotroph (Zhao et al., 2021). In our study, DOC generally increased with altitude and reached its maximum at the highest altitude (E1485), where the type II methanotroph dominated at the higher altitude. Although the concentrations of type I and type II methanotroph biomarkers were both significantly correlated with the altitude, the effect of location on the concentrations of the two types of methanotroph biomarkers exceeded that of altitude (Table 2 and Figure 2).

### Characteristics of Methanotroph Metabolism Along the Altitude Gradient

The δ^13^C values of type I and type II methanotroph biomarkers differed significantly along the altitude gradient (Figure 4). The δ^13^C values of type I methanotroph biomarkers were more negative and the isotope composition ranged widely (Figure 4). Type I methanotrophs primarily used CH_4_ with extremely negative isotope values (–65‰ ≤ δ^13^C ≤ –50‰) as an energy source (Whiticar et al., 1986), and type I methanotrophs combine CH_4_ into the membrane and use CH_4_ for assimilation and energy generation. The CH_4_ is finally converted into CO_2_ and released into the atmosphere (Holmes et al., 1999; Bull et al., 2000). Meanwhile, the type II methanotrophs assimilated about half of the carbon from CH_4_, and the rest was derived from CO_2_ (Hanson and Hanson, 1996; Holmes et al., 1999). Similarly, the type II methanotrophs were also found to assimilate CH_4_ and CO_2_ to form CO_2_ and release it into the atmosphere in the subtropical forests (Zhou et al., 2021). Interestingly, the type II methanotroph biomarker in E430 was more negative than in other sites, it may be because the abundant TN and TP in soils promoted the utilization rate of CH_4_, and thus increased the microbial activity and carbon isotope fractionation (Steudler et al., 1989). This phenomenon also confirmed the accuracy of 18:1ω7c as the methanotroph biomarker in the sedge peatlands.

Compared with the type II methanotrophs, the change of δ^13^C values of type I methanotrophs was more significant along the altitude gradient. The δ^13^C value of type I methanotroph biomarker was lowest in E683, this site may produce the most abundant CH_4_ used by methanotrophs due to the difference in vegetation (Chen et al., 2009), water table (Kim et al., 2011; Tian et al., 2015; Shao et al., 2017), and temperature (King and Adamsen, 1992; Sitaula et al., 1995). Abundant CH_4_ and O_2_ can provide a suitable environment for the growth of the methanotrophs and promote CH_4_ oxidation activity. Less than maximum fractionation is observed when cells are grown with reduced CH_4_ availability (Summons et al., 1994). The obvious change in the δ^13^C values of type I methanotroph biomarkers with the altitude gradient may also be due to their rapid response to the environmental changes, which has been found by other studies (Henckel et al., 2000). Meanwhile, former studies found that methanotrophs were closely related to CH_4_ concentration in the atmosphere (Chen et al., 2008). In our study, combining the concentrations and natural ^13^C content of the methanotroph biomarkers, we found that the concentrations of type I methanotrophs using CH_4_ as the only carbon source decreased with the increase of altitude, which further indicated that the ability of CH_4_ consumption at higher altitudes decreased. The peatlands at a higher altitude might retain more unstable CH_4_ than at a lower altitude.

## Conclusion

This is the first research on the concentrations and metabolic characteristics of methanotrophs along an altitude gradient in the northern peatlands. We found that the patterns of type I and type II methanotroph biomarkers differed significantly along the altitude gradient. RDA analyses indicated that the type I methanotroph biomarkers were positively correlated with TP and TN, and the type II methanotroph biomarkers were positively correlated with DOC. Compared with type II, the change in the δ^13^C values of type I methanotroph biomarkers was more obvious as the altitude increased, which indicated their different preferences for substrate utilization. Our study laid the foundation for the study of CH_4_ metabolism in peatlands along the altitude gradient. Further research combined with CH_4_ flux and 16S rRNA sequencing data is required to better understand the biogeochemical process of methanotrophs in peatlands under the warming climate.

## Data Availability Statement

The original contributions presented in the study are included in the article/supplementary material, further inquiries can be directed to the corresponding author.

## Author Contributions

MZ, MW, and GW designed the study. MZ, MW, YZ, and MJ performed the field investigation and collected the data. MZ and GW conducted the statistical analysis and wrote the manuscript. All authors reviewed and contributed to the manuscript writing.

## Conflict of Interest

The authors declare that the research was conducted in the absence of any commercial or financial relationships that could be construed as a potential conflict of interest.

## Publisher’s Note

All claims expressed in this article are solely those of the authors and do not necessarily represent those of their affiliated organizations, or those of the publisher, the editors and the reviewers. Any product that may be evaluated in this article, or claim that may be made by its manufacturer, is not guaranteed or endorsed by the publisher.
